# Early Graft Reperfusion and Arrhythmias After Coronary Artery Bypass Grafting

**DOI:** 10.7759/cureus.64285

**Published:** 2024-07-10

**Authors:** Joona Keronen, Tuomas Huttunen, Ari Mennander

**Affiliations:** 1 Cardiothoracic Surgery, Tampere University Heart Hospital, Tampere, FIN; 2 Anesthesiology and Critical Care, Tampere University Heart Hospital, Tampere, FIN; 3 Cardiothoracic Surgery, Tampere University, Tampere, FIN

**Keywords:** surgical technique, ischemia-reperfusion injury, arrhythmia, coronary artery bypass grafting (cabg), left internal thoracic artery

## Abstract

Background

Arrhythmia after coronary artery bypass grafting (CABG) may occur immediately after the abrupt onset of reperfusion via all coronary bypass grafts simultaneously. We investigated whether early reperfusion of the left anterior descending coronary artery before weaning from cardiopulmonary bypass would decrease the frequency of early arrhythmias after CABG. We compared patients undergoing release of the left internal thoracic artery (LITA) graft flow before versus after aortic declamping during CABG.

Methodology

In total, 109 consecutive patients undergoing CABG were retrospectively analyzed. The heart rhythms after CABG of 46 patients with flow release from LITA before aortic declamping (study group) were compared with 63 patients with complete onset of reperfusion of all coronary bypass grafts simultaneously after aortic declamping (controls). Early arrhythmias were recorded and included atrial fibrillation, ventricular tachycardia, ventricular fibrillation, and arrhythmias necessitating temporary pacemaker support.

Results

Early arrhythmias occurred in seven out of 46 study group patients with the early release of LITA graft flow compared with 21 out of 63 controls (15.2% vs. 33.3%, p = 0.033). Creatine kinase-myocardial band levels were lower in the study group than in the controls (27.5 ± 58.4 vs. 33.0 ± 48.0, p = 0.004, respectively). Sinus rhythm was achieved in all but three patients before extubation including two in the study group and one in the controls.

Conclusions

The simple maneuver of releasing LITA graft flow before aortic declamping during CABG allows gradual reperfusion of the myocardium and may ensure early rhythm control.

## Introduction

Coronary artery bypass grafting (CABG) using cardiopulmonary bypass is a well-established surgical means to ensure coronary artery circulation in patients with coronary artery disease [1]. The early restoration of coronary artery circulation after CABG enables early recovery, but postoperative arrhythmias may delay recovery [2]. Early recovery is essential for excellent patient satisfaction and fast-track postoperative treatment, including cardiac rhythm control [2,3]. During CABG, prevention of reperfusion ventricular arrhythmias after the release of aortic cross-clamping may be managed medically [4,5]. Cardioversion or defibrillation together with immediate pacemaker support are additional means to secure sinus or stable rhythm early after surgery.

We routinely use cold crystalloid cardioplegia that is given antegrade or retrograde every 10 to 15 minutes during CABG and apply warm cardioplegia immediately before release of the aortic cross-clamp. However, we and others have encountered a few arrhythmias immediately after weaning from cardiopulmonary bypass, although we have rarely experienced permanent ventricular fibrillation [6,7]. Overall, postoperative outcomes after CABG are usually uneventful, but only after intensive rhythm control in our intensive care unit for approximately 24 hours.

Relatively little has been discussed on immediate preventive rhythm control after CABG and reperfusion ventricular fibrillation [3,7]. An abrupt blood flow to coronary arteries may lead to increased cardiac filling pressure and induction of arrhythmias [4]. Ventricular arrhythmias have been associated with oxidative stress reactions triggered after cardiac ischemia [8] and uncontrolled reperfusion with abrupt restoration of extracellular electrolyte balance [9]. We hypothesized that prevention of arrhythmias after cardiopulmonary bypass may be associated with early flow restoration of coronary artery circulation. We retrospectively reviewed the early outcomes of our recent patients with and without early release of coronary artery blood flow from the left internal thoracic artery (LITA) graft during CABG.

## Materials and methods

We compared two patient groups that underwent CABG between January 2019 and May 2023 by a single surgeon in Tampere University Heart Hospital. Upon technical fluency during surgery, CABG was performed using similar techniques in all patients, except that after May 20, 2021, flow from the LITA graft was released after performing all distal anastomoses instead of releasing blood flow to all grafts simultaneously after aorta declamping. Data on early rhythm control and patient outcomes were collected from patient charts after CABG. This observational study was an example of a contemporary cross-sectional study cohort from real life. The study design included a representative random sampling of consecutive patients operated on by a single surgeon. The retrospective study approach was approved by the institutional review board (Ethical Committee of the Tampere University Hospital, Tampere, Finland, R23028). The need for informed consent was waived, and the study conformed to the ethical guidelines of the Declaration of Helsinki.

Surgical technique

**Figure 1:**
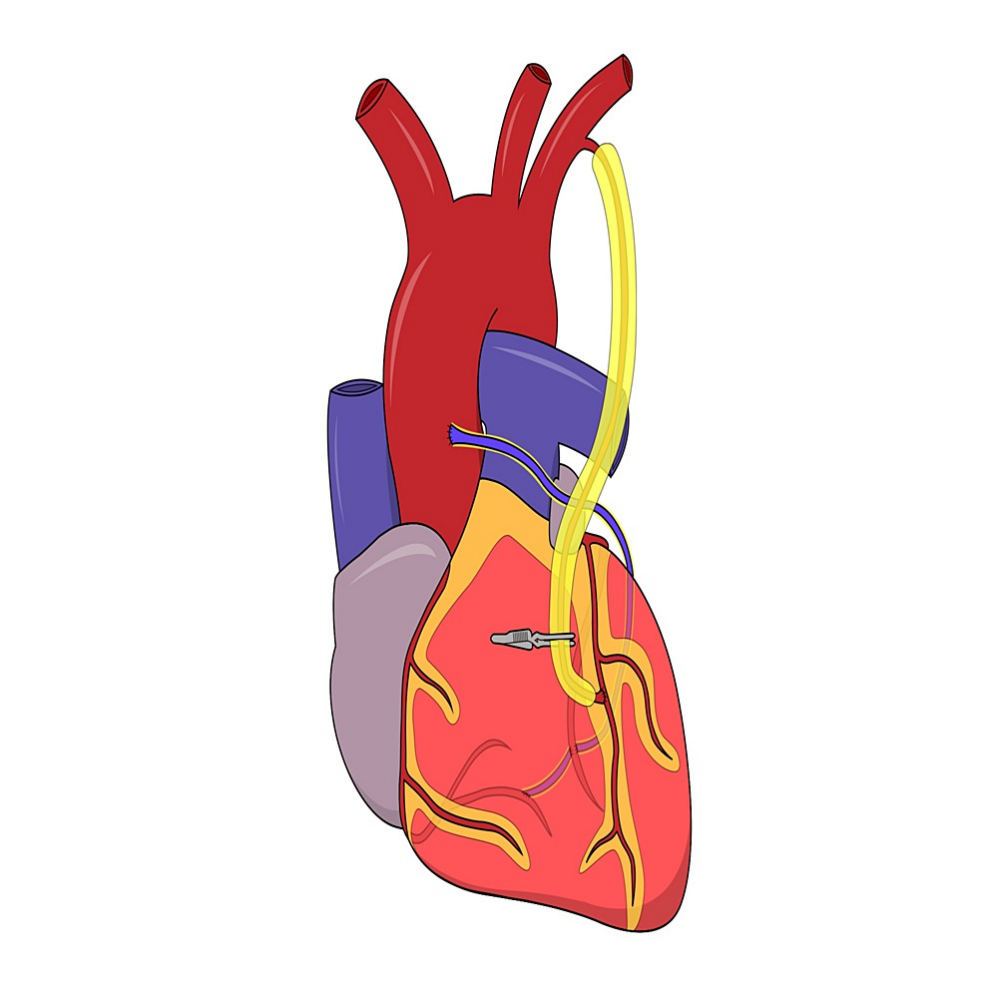
Schematic presentation of the heart after completion of coronary artery bypass grafting with an arterial clamp of the left internal thoracic artery. Note the arterial clamp of the left internal thoracic artery is either released early versus after completion of the proximal graft anastomose to the aorta in the study group versus controls, respectively. Figure created by the authors.

CABG was performed in a traditional manner using cardiopulmonary bypass. While the venous and/or radial artery grafts were harvested, sternotomy was performed, the patient was heparinized, and the LITA was prepared in a pedicled no-touch fashion leaving both adjacent veins intact to support and protect the artery graft itself. Cardiopulmonary bypass was initiated, an aortic cross-clamp was applied, antegrade blood cardioplegia was administered every 15 minutes, and the distal anastomoses were performed using a 7-0 surgical suture line. Revascularization of the main areas of the myocardium nourished by the three main coronary artery branches, namely, the right coronary artery, the left circumflex coronary artery, and the left anterior descending artery (LAD), was performed in a step-by-step manner, according to the preoperative coronary artery angiogram. Usually, the vein graft was utilized as a snake graft providing an end-to-side anastomose to the distal part of the right coronary artery, while a side-to-side sequential anastomose was performed to the left circumflex coronary artery or one of its left obtuse marginal coronary artery. The LITA graft was anastomosed to the LAD. A baby duck applied on the LITA provided surgical visualization while performing the distal anastomose of the graft to the LAD which was released thereafter (study group). Therefore, we applied an early 10-minute reperfusion of the heart via the LITA graft in the study group. In controls, the baby duck was kept closed until all distal and proximal anastomoses of the vein and/or radial artery were accomplished to the side of the ascending aorta, and reperfusion of the heart occurred simultaneously throughout all grafts and native coronary arteries upon releasing the aortic cross-clamp (Figure 1).

Rhythm control

After completion of all anastomoses, warm antegrade cardioplegia was administered for three minutes, and aortic declamping was performed. Cardiac rhythm was monitored and intravenous lidocaine/amiodarone was administered if ventricular tachycardia or fibrillation was observed. Defibrillation was done if arrhythmias persisted for more than two minutes. The aim was to achieve a constant rhythm, preferably a sinus rhythm. An epicardial temporary pacemaker with a fine needle was implanted through the right ventricular epimyocardium in all patients. Two mediastinal drainage tubes were left before applying sternum wires and closing the wound in a usual manner. The patients were transferred to the intensive care unit for subsequent rhythm monitoring and recovery. Early arrhythmias were recorded and included atrial fibrillation, ventricular tachycardia, ventricular fibrillation, and arrhythmias necessitating temporary pacemaker support. Routine laboratory tests such as creatinine kinase (CK, U/L), creatinine kinase-myocardial band fraction (CK-MB, µg/L), and leukocytes (10^9^/L) were compared between the patents the following day after surgery.

Statistical analyses

Statistical analyses were done using SPSS version 28.0 (IBM Corp., Armonk, NY, USA) program. Categorial values are expressed as absolute values and percentages, and continuous values are shown using means and standard deviations (SDs). Comparisons of the groups were made using the chi-square and the non-parametric Mann-Whitney U-test, when appropriate. A p-value <0.05 was considered significant. Post hoc statistical power calculation was set to display the 95% confidence interval and performed with statistical software (ClinCalc, clincalc.com). For the effect size (group incidences of 26.1% vs. 57.1%), patient number (46 and 63), alpha (0.050, two-tailed), statistical power was 0.91.1%.

## Results

Patient characteristics

Patient characteristics are shown in Table 1. There were 46 patients with early reperfusion of the LITA graft during CABG (study group) and 63 controls with reperfusion of all grafts simultaneously after aortic declamping. In total, there were 90 (82.6%) male patients. The mean age for the patients in the study group was 68.5 years (SD = 9.1), while it was 69.2 years (SD = 7.1) for controls. A family history of coronary artery disease, dyslipidemia, hypertension, smoking, and diabetes was equally represented in both groups. Euroscore II was 7.7 and 8.4 in the study group and controls, respectively.

**Table 1:** Patient characteristics. SD = standard deviation

	Study group	Controls	P-value
Age (years, SD)	68.5 (9.1)	69.2 (7.1)	0.949
Gender (male)	39 (84.8%)	51 (81.0%)	0.603
Family history of coronary artery disease	15 (32.6%)	28 (44.4%)	0.212
Dyslipidemia	39 (84.8%)	48 (76.2%)	0.270
Hypertension	40 (87.0%)	48 (76.2%)	0.159
Diabetes	21 (45.7%)	27 (42.9%)	0.772
Ejection fraction (%, SD)	51.3 (14.5)	53.9 (10.5)	0.524
Body mass index (SD)	28.5 (4.1)	27.2 (4.2)	0.056
Acute myocardial infarction	2 (4.3%)	6 (9.5%)	0.306
Euroscore II (SD)	7.7 (10.7)	8.4 (12.8)	0.927
Creatine (µmol/L, SD)	82.7 (22.0)	85.8 (24.8)	0.493
Smoking	8 (17.4%)	10 (15.9%)	0.978
Not smoking	25 (54.3%)	35 (55.6%)	
Previous smoking	13 (28.3%)	18 (28.6%)	

Surgical details

As shown in Table 2, there were no differences in surgical details among the groups. The number of grafts and surgical and cardiopulmonary times did not differ among the patient groups.

**Table 2:** Surgical details. SD = standard deviation

	Study group	Controls	P-value
Elective	19 (41.3%)	24 (28.1%)	0.287
Urgent	19 (41.3&)	20 (31.7%)	
Emergency	8 (17.4%)	19 (30.2%)	
Number of grafts (SD)	2.83 (0.6)	3.05 (0.8)	0.173
Cross-clamp time (minutes, SD)	81.9 (16.8)	83.0 (19.5)	0.910
Cardiopulmonary bypass time (minutes, SD)	102.6 (21.3)	109.0 (24.2)	0.191

Early postoperative outcome

The different immediate postoperative arrhythmias are shown in Table 3.

**Table 3:** Immediate postoperative cardiac rhythms. n = number of patients

		Study group (n = 46)	Controls (n = 63)
Sinus rhythm		16	12
Transient rhythms		23	30
	Asystole	3	15
	Junctional rhythm	4	7
	Conpensational rhythm	10	7
	Wide complex rhythm	3	1
	Narrow complex rhythm	3	0
Early arrhythmias		7	21
	Pacemaker rhythm	3	13
	Atrial fibrillation	1	1
	Ventricular fibrillation	2	4
	Ventricular tachycardia	1	3

Only seven patients out of 46 in the study group experienced early arrhythmias immediately after weaning from cardiopulmonary bypass compared with 21 out of 63 controls (15.2% vs. 33.3%, p = 0.033, respectively) (Table 4). Immediate pacemaker support, cardioversion, or defibrillation was needed in little more than a quarter of the study group patients compared with more than half of the controls (26.1% vs. 57.1%, p = 0.001, respectively). For the effect size (group incidences of 26.1% vs. 57.1%), patient number (46 and 63), alpha (0.050, two-tailed), statistical power was 0.91.1%. The plasma value of CK-MB was lower in the study group patients compared with controls (27.5 ± 58.4 µmol/L vs. 33.0 ± 48.0 µmol/L, p = 0.004, respectively). CK-MB to CK ratio was lower in the study group patients compared with controls (0.043 ± 0.021 vs. 0.053 ± 0.024, p = 0.009). Sinus rhythm was achieved in all but three patients before extubation, including two in the study group and one in the controls. Altogether, two patients experienced strokes, there were three mediastinitis, one patient underwent dialysis, and four patients died, though there were no differences among these early outcome events among the patient groups.

**Table 4:** Early postoperative outcomes. * = see Table 3; CK = creatinine kinase; CK-MB = creatinine kinase myocardial band fraction; SD = standard deviation

	Study group	Controls	P-value
Early arrhythmia*	7 (15.2%)	21 (33.3%)	0.033
Immediate pacemaker support, cardioversion, or defibrillation	12 (26.1%)	36 (57.1%)	0.001
CK (U/L, SD)	555.7 (732.2)	561.93 (414.5)	0.644
CKMB (µg/L, SD)	27.5 (58.4)	33.0 (48.0)	0.004
Leukocytes (10^9^/L, SD)	11.5 (3.9)	12.4 (5.0)	0.674
CK-MB/CK ratio	0.043 (0.021)	0.053 (0.024)	0.009
Stroke	2 (4.3%)	0 (0%)	0.095
Mediastinitis	2 (4.3%)	1 (1.6%)	0.384
Dialysis	0	1 (1.6%)	0.391

## Discussion

This retrospective study suggests that early coronary artery reperfusion by releasing flow to the LAD through the LITA before aortic declamping ensures early cardiac rhythm control and decreases the risk of ventricular arrhythmias immediately after surgery. CABG was performed using similar techniques in all patients except that flow from the LITA graft was either released before performing the proximal anastomose while the aorta remained cross-clamped (study group) or after warm cardioplegia and aortic declamping (controls).

Based on our traditional institutional protocol before May 20, 2021, graft flow release was allowed only after completion of all surgical steps during warm cardioplegia and aortic declamping (controls). This strategy ensured a bloodless surgical field, thus affording a good technical milieu for smooth surgery. However, we often experienced a few persistent ventricular arrhythmias leading to further medication and defibrillation of the heart before allowing weaning from cardiopulmonary bypass.

The means of early release of antegrade blood flow through the LITA during routine CABG was initially launched in our institution to ensure unrestricted flow to the grafted LAD (study group). Although routine flow measurements were also performed during CABG, it was felt that the interpretation of the graft flow curve and pressure index values were not always without difficulties. We adapted the early temporary means to release LITA flow to the LAD to visualize unrestricted blood flow to the anterior part of the myocardium to facilitate fluent surgery and perioperative management. It was soon acknowledged that reperfusion of LAD entailed early myocardial rewarming with often a stable cardiac rhythm, though the warm cardioplegia had not yet even been administered. Indeed, it is unclear whether the use of terminal warm blood cardioplegia decreases the incidence of arrhythmias after cardiac surgery [7]. Therefore, we left the LITA graft unclamped and proceeded to finish CABG while the aortic cross-clamp was still in place for the approximately 10 minutes needed to finalize the proximal anastomose of another graft to the side of the ascending aorta.

Several experimental models have been suggested to study cardiac reperfusion after coronary artery occlusion and controversial associations with ventricular arrhythmias have been discussed [10,11]. The incidence of reperfusion arrhythmias depends on the experimental species [10] and on the experimental set-up itself [12]. Alteration of myocardial microcirculation may impact early outcomes after cardiac surgery [13-15]. In our study, early release of ischemic myocardial CK-MB values was lower in the study group compared with controls. Statistical differences were not observed in either plasma leukocyte count, aortic cross-clamp, and cardiopulmonary bypass times among the groups. As sinus rhythm was achieved in all but three patients before extubation, the long-term significance of gaining immediate rhythm control remains to be explored.

Immediate rhythm control may be challenging after CABG, although early arrhythmia may be treated using immediate pacemaker support, cardioversion, or defibrillation, in addition to medication [16,17]. Early arrhythmias may predispose to inadequate graft blood flow and circulation, thus increasing cardiac filling pressure and volume overload after CABG [18]. Even temporary hemodynamic instability may predispose to the increased inflammatory response of cardiac tissue, while the pathogenesis of arrhythmias may be associated with the induction of inflammation after cardiac surgery [19,20]. On the other hand, the relatively small number of patients in this study prevents deducing definitive conclusions, e.g., from postoperative leukocyte count and patient outcome. As patient characteristics and surgical urgency were comparable among the patients, the even moderate release of cardiac enzymes may reflect temporal cardiac insult after CABG [21]. It is tempting to suggest that early gain of hemodynamic and rhythm control may at least ease patient recovery.

Limitations

The limitations of this study include the observational and retrospective approach with a small number of patients without long-term follow-up. The onset of early arrhythmia occurred while the surgical wound was still open after aortic declamping and other causes of postoperative arrhythmia were not studied. On the other hand, the patient characteristics were similar among the groups, and surgery was performed consistently by a single surgeon in a real-world clinical set-up. Although power calculation of even up to 91.1% for the release of LITA flow before aortic declamping indicates that gradual reperfusion may impact the immediate need for arrhythmia treatment, it is pertinent to warrant further clinical evidence.

## Conclusions

Abrupt myocardial reperfusion upon releasing coronary artery flow via all grafts simultaneously after aortic declamping may exacerbate myocardial ischemia-reperfusion. Instead, early restoration of stabile sinus rhythm may be achieved sooner. Our study suggests that, due to the observational limitations and selection bias, gradual reperfusion by releasing LITA blood flow before weaning from cardiopulmonary bypass may decrease the risk of early ventricular arrhythmias. A randomized controlled trial is needed to confirm these findings.
